# Application of digital child mental health training to improve capacity in majority world countries: Professional perspectives from Turkey and Pakistan

**DOI:** 10.1177/13591045211046809

**Published:** 2021-09-25

**Authors:** Panos Vostanis, Seyda Eruyar, Sajida Hassan, Reem AlOwaybil, Michelle O’Reilly

**Affiliations:** 1 Department of Neuroscience, Psychology and Behaviour, 4488 University of Leicester, Leicester, UK; 2 Department of Psychology, 226846 Necmettin Erbakan University, Konya, Turkey; 3 Hussaini Foundation, Karachi, Pakistan; 4 Department of Sociology, 98072 University of Leicester, UK

**Keywords:** child mental health, digital, training, capacity building, majority world, low-income countries

## Abstract

Digital technology offers opportunities for child mental health capacity building, which is a priority for Majority World Countries (MWC). The aim of this study was to explore the experiences and perspectives of professionals from different disciplines in Turkey (*n*=12) and Pakistan (*n*=15), who had completed a two-module digital trauma-informed programme on enhancing practice skills and instigating systemic changes. Interview data were analysed through a coding thematic approach. Participants especially valued the interdisciplinary and holistic approach of the training, and its proposed scaled service model. Digital training, particularly in blended format, can enhance reach and capacity in MWC low-resource settings.

## Introduction

It is well established that children living in Majority World Countries (MWC) have high levels of unmet mental health needs. These needs are especially pronounced in areas of post-conflict and disadvantage (World Health Organisation, 2016). Reasons involved include stigma of mental health, caregiver disengagement, lack of culturally adapted interventions and limited specialist resources (Patel et al., 2018). Mental healthcare is often delivered by agencies such as schools, non-governmental organisations (NGOs), community groups and volunteers. Service provision is often combined with other functions such as child protection, health promotion and life-skills training (Van Ginneken et al., 2013). Capacity building is, therefore, crucial in improving service provision, to maximise impact (World Health Organisation, 2013). Several child mental health programmes have been reported for primary healthcare professionals, teachers or lay community workers (Murray et al., 2011). Such training was found to increase knowledge and competencies, although this needed to be accompanied by systemic changes to tackle barriers such as stigma.

Digital technology has enhanced opportunities in managing mental health problems in primary health settings or schools (Long et al., 2018). Despite the global ‘digital divide’, the gap is closing over time (Naslund et al., 2017). Web-based training for teachers in Brazil led to improved knowledge (Pereira et al., 2015), whilst primary healthcare professionals in India reported reduced stigmatising attitudes (Muke et al., 2020). Nevertheless, there is little knowledge regarding how digital training can contribute to capacity building in MWC, in relation to design, content and delivery. This gap informed the rationale for this study.

## Method

Building on available evidence, the aim of the digital training described below was to equip professionals in contact with children with mental health needs in MWC with additional knowledge and skills to improve their practice and to initiate wider service changes. Providers are educationalists, psychologists, social workers, physicians, NGO workers and volunteers (Patel et al., 2018). The training was not designed to address capacity, but rather to help inform planning. The aims of this study were to establish the perspectives of professionals from Turkey and Pakistan on which training components could be useful for future capacity building, and how web-based delivery could be incorporated. These aims were addressed through the following research questions:

1. How can mental health training be augmented to facilitate capacity building in resource-constrained settings?
2. Which training components are relevant for practice in real-world contexts?
3. How could digital platforms contribute to capacity building?

### Context and participants

The two Asia MWC reflect different economic status on the MWC spectrum (OECD, 2016) and extent of mental health services (World Health Organisation, 2016). They have similarities in terms of increasing child population, inequalities, religion, family structures and community supports (Shujaat, 2015; Turkish Statistical Institute (TurkSat), 2020). This aided the understanding of how the research questions are addressed in these contexts, rather than a comparative per se design. In each country, a host non-governmental organisation (NGO) facilitated recruitment of professionals. The NGOs were partners in a global network described in Vostanis (2019). Through local networks, each NGO invited professionals and volunteers operating in their proximity and supporting children in relation to their mental health. All professionals had a first degree and several had a Masters qualification (mostly in psychology), although this is not an essential requirement to practice, as there are no formal qualifications in child mental health. They ranged in experience and discipline, and often combined their agency role with private practice (Table 1). We adopted a purposeful sampling frame until the selected sample reached thematic saturation (Francis et al., 2010). In total, 12 professionals participated in Turkey and 15 in Pakistan.

**Table 1.** Participants’ profile.

Participant	Age	Gender	Graduate qualification	Postgraduate qualification	Past experience	Current occupation
Turkey (*n*=12)						
1	27	Male	Special education	Special education and psychodrama training	NGO special education teacher	Research assistant in special education, and special needs NGO volunteer
2	26	Male	Special education	Special education	NGO special education teacher	Research assistant in special education, and special needs NGO volunteer
3	30	Female	Psychology	Clinical psychology	Private psychotherapy practice	NGO worker for refugee children (psychosocial support and psychotherapy)
4	35	Female	Psychology	Clinical psychology	Private psychotherapy	NGO worker for refugee children (psychosocial support and psychotherapy)
5	25	Female	Counselling and guidance	—	NGO volunteer for children in disadvantaged areas	NGO counsellor for refugee children (psychoeducation and psychosocial support)
6	27	Female	Psychology	Clinical psychology	NGO volunteer for children in disadvantaged areas	NGO worker for children with Down’s syndrome (family support and community integration)
7	32	Female	Counselling and guidance	—	NGO volunteer for children in disadvantaged areas	NGO counsellor for refugee children (psychoeducation and psychosocial support)
8	35	Male	Social work	Social psychology	NGO volunteer for children in disadvantaged areas	Child protection officer for refugee children
9	29	Female	Management	—	—	NGO manager
10	27	Female	Counselling and guidance	—	NGO volunteer for children in disadvantaged areas	NGO counsellor for refugee children (psychoeducation and psychosocial support)
11	35	Female	Psychology	Clinical psychology	Private psychotherapy practice	NGO worker for refugee children (psychosocial support and psychotherapy)
12	32	Female	Pre-school education	Education science	Special education teacher	Pre-school teacher in disadvantaged area with refugee population
Pakistan (*n*=15)						
1	27	Male	Education	Educational leadership and training	NGO teacher	Teacher and teacher trainer
2	36	Female	Education	Education and teacher training	Teacher	Academic
3	30	Female	Psychology	Educational psychology	School counsellor	Psychologist at inclusive care centre
4	32	Female	Psychology	Educational psychology	School and college counsellor	Counsellor in private setting (online and home-based)
5	24	Female	Psychology	Clinical psychology	School and college counsellor	Counsellor in private setting (online and home-based)
6	38	Female	Education	Educational leadership and management	Special education teacher	Head teacher in disadvantaged area
7	24	Female	Criminology	Criminology	NGO volunteer for children in disadvantaged areas	Social worker/volunteer at juvenile correctional centre
8	23	Female	Sociology	Sociology and Islamic studies	Islamic education teacher and religious scholar	Islamic education teacher and religious scholar
9	25	Female	Islamic studies	Arabic and Islamic studies	Islamic education teacher and religious scholar	Islamic education teacher and religious scholar
10	32	Female	Islamic studies	Islamic studies and early childhood education	Islamic education teacher and religious scholar	Islamic education teacher and religious scholar
11	25	Female	Psychology	Applied psychology	Youth motivational speaker and trainer	Lead trainer on youth and female empowerment
12	32	Female	Psychology	Counselling and guidance	Counsellor in NGO school	Counsellor in private setting (support groups for women)
13	22	Female	Psychology	Applied psychology	NGO youth volunteer	NGO youth volunteer
14	23	Female	Psychology	Clinical psychology	NGO youth volunteer	NGO youth volunteer
15	24	Female	Home economics	Food and nutrition	NGO youth volunteer	NGO youth volunteer

### Digital training modules

The overall purpose of the training is to enhance the competencies of professionals in understanding the complex mental health needs of children who experience traumatic experiences, recognise and address those needs through evidence-based interventions and bring systemic changes to services and communities (Vostanis, 2019). This consists of two modules based on digital adaptation of face-to-face training, which was previously implemented and evaluated in several MWC, including Turkey and Pakistan. The web-based modules were developed by a network of child mental health professionals from MWC and the UK. Their digital adaptation was made by the first author in collaboration with a digital platform developer, who also hosts the modules. The focus is on children and young people between 3 and 18 years, with a child development section included at the beginning of module 1, and developmentally appropriate knowledge and exercises incorporated throughout the training.

**Figure 1.**
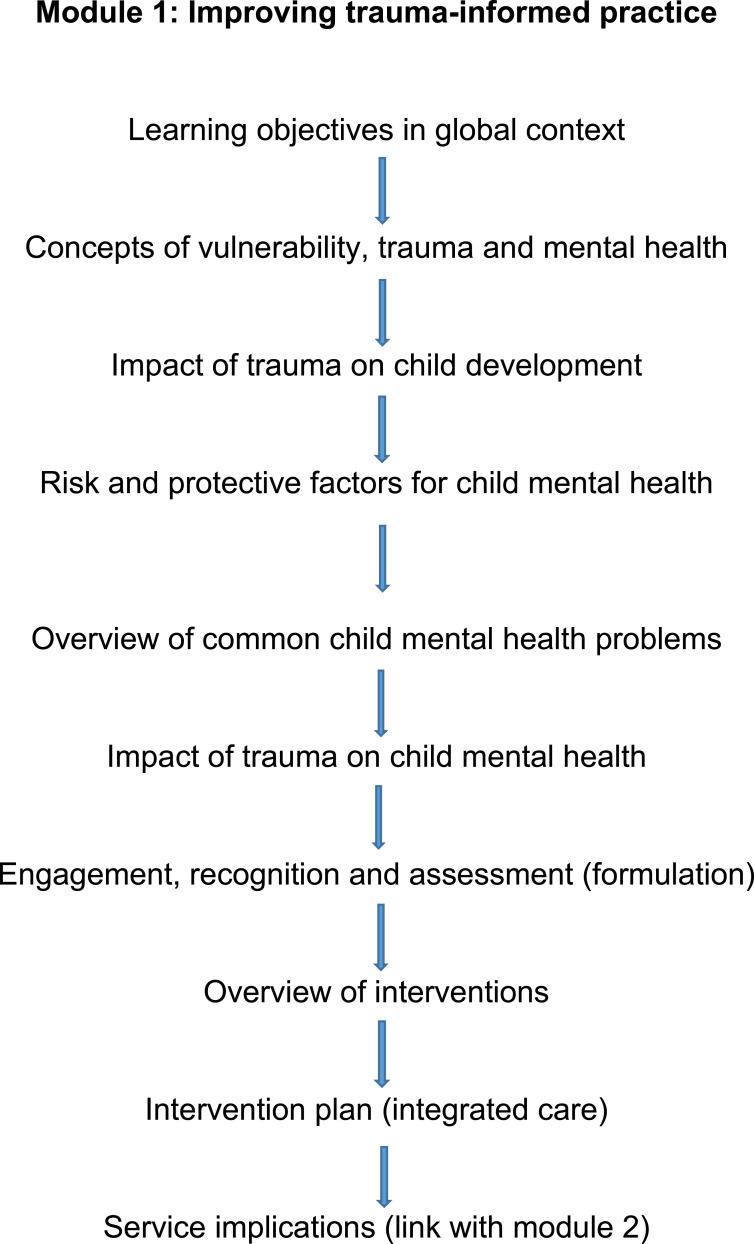
Module 1: Improving trauma-informed practice.

**Figure 2.**
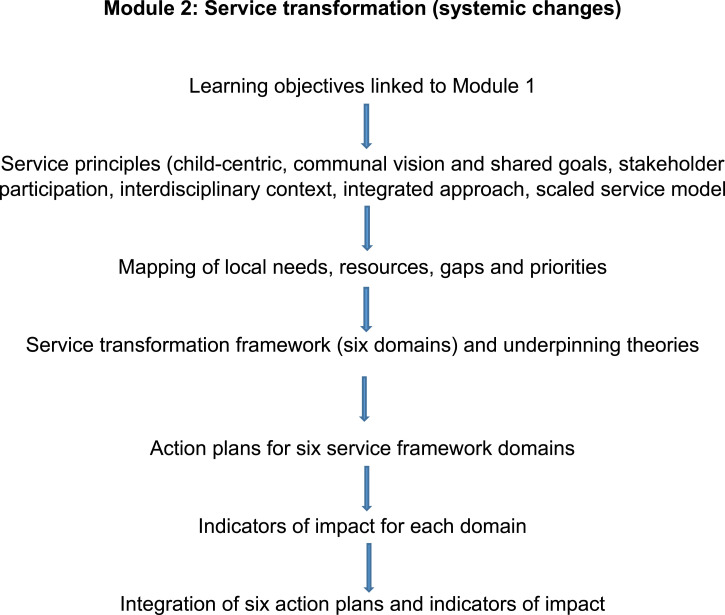
Module 2: Service transformation (systemic changes).

Each module consists of approximately 8–10 topics broken into 50 short sections, with key information. The key topics for each module are presented sequentially in Figures 1 and 2, respectively. This information is interspersed with self-reflection, video- and case-based exercises. Participants are encouraged to consider these exercises before continuing the training. Some exercises, especially in formulating service action plans, should preferably be completed with their colleagues. Overall, each non-facilitated module can be completed within 1–2 hours, although it is recommended to practice and revisit learning points over a period of 2 weeks. The digital adaptation and hosting of the modules incurred small costs, which were covered by a research fund. No booster training or facilitation was built in this study because of capacit*y.* The same modules were used in both countries. However, all exercises were based on participants’ caseload and local/national contexts, especially in module 2, where they were encouraged to consider local/national policy.

### Module 1: Improving trauma-informed practice

This module aims to improve mental health skills within existing roles, and to build interprofessional values. Its specific objectives are to enhance knowledge and skills on how children’s development and mental health are affected by multiple risk factors, identify factors which promote children’s resilience, recognise common child mental health problems and plan interventions that take into consideration other professionals and agencies in their area (Vostanis et al., 2019b) (Figure 1).

### Module 2: Service transformation

**Figure 3.**
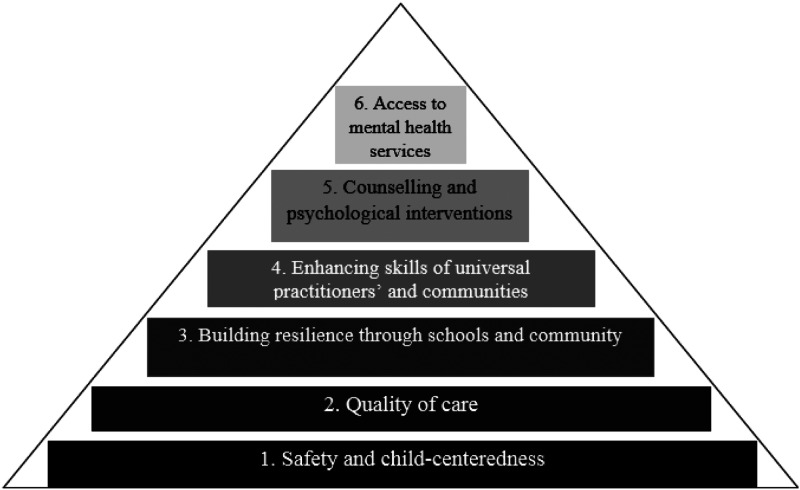
Service transformation framework (Vostanis et al., 2019a).

The second module aims at systemic improvements in service provision that go beyond individual practice. Its objectives are to establish interprofessional networks, map current strengths and gaps in resources, co-produce action plans with local stakeholders and implement these action plans in a target area (Vostanis et al., 2019a) Figure 2). Participating agencies work together along six domains of a service transformation framework informed by child mental health literature in MWC, socioecological systems theory (Bronfenbrenner, 1979) and the scaled service model (World Health Organisation, 2016). The six domains aim to improve children’s safety, support parents, promote children’s resilience through schools and communities, upskill professionals and community volunteers, enhance provision of counselling and improve access to mental health services (Figure 3). Action plans need to be realistic, achievable, accountable, measurable and short-term (3–6 months), albeit within a strategic direction. Participants are encouraged to co-produce indicators of impact. The digital format is like the first module, that is, it includes case-based discussion and reflexivity exercises. In addition, we developed video material with children, parents and professionals from Turkey and Pakistan on how they could benefit from services on each domain.

### Research procedure

The study received approval by the University of Leicester Research Ethics Committee in the UK. We consulted on research ethics jurisdictions in both countries, and there was no appropriate ethics board, especially for research through non-governmental organisations (NGOs), which acted as gatekeepers. Participants provided written informed consent, following which they completed the two digital modules and remotely attended semi-structured interviews within 2 weeks. Each interview followed a semi-structured guide of three sections on their role (agency, context, experience and previous training); challenges (knowledge, skills, service, resource, organisational and cultural) and perspectives of training in relation to capacity building (relevance to role and sociocultural context, learning style, digital platform and recommendations).

### Analysis

To promote a person-centred focus that accounted for the existing knowledge base, we utilised the codebook thematic approach, as this is a category-based coding process that prioritises participant narratives (Braun & Clarke, 2019). Three coders independently worked through the data and via a verification process and open dialogue, merged the data into a final coding frame. This process resulted in three overarching themes and subthemes in relation to the research questions (Table 2).

**Table 2.** Emerging themes and subthemes on professionals’ perspectives.

Themes	Subthemes
Relevance to staff role	Training needs
	Linking theory and evidence with practice
	Training in an interdisciplinary context
	Influence of religion
Appraisal of training content, format and delivery	Understanding complexity of needs
	Relating to non-specialists
	Hierarchical and holistic approach
	Mode of delivery
Value of understanding systems	Changing practice
	Scaled service model
	Implications for service improvement

## Results

### Theme one: Relevance to staff role

In articulating their training needs, participants initially considered their qualifications, experience and previous training. Typically, this was theoretical and at undergraduate level, followed by short courses, mainly on specific training modalities such as cognitive-behavioural therapy (CBT). Recognised needs were in communication, detection of mental health problems, and trauma-informed practice.

“…In that part, we know the psychological processes, there are certain reactions in the case of trauma, but how it progresses in a more specific group or more cases and situations should be addressed.” Counsellor, Turkey

“One needs to be properly trained in the specific field for positive and effective outcome, and in order to actually help because, as I said, it is very risky and dangerous to work in this area. If any of the body parts is broken, at least the brain is functioning, but working with someone’s mind is really dicey”. Religious scholar, Pakistan

Some participants appreciated the child-centric philosophy and recognised the importance of awareness and training in child protection and children’s rights. Participants argued that many interventions were overly western and not adapted to different cultural contexts, the training thus required more culturally appropriate case examples and content. Training for trainers was identified as a neglected area.

“There is a legal procedure for early marriage and child labour, I would like to learn more about them…I try to concentrate on more practical training to improve myself, what can I use when conducting individual interviews with children, what can I add when doing group work?.” Counsellor, Turkey

Participants discussed in depth the importance of theory, evidence and gaining their own experience. Several theories were mentioned as being relevant to their practice such as humanistic, attribution, attachment and Maslow’s hierarchy of needs. A few professionals added the value of evidence in informing their choice of interventions. Most participants valued learning in the field, applying and testing theoretical knowledge. Continuity of training through supervision was viewed as important in sustaining benefits.

“Something which really struck me was the (Figure 3) model, the pyramid. It was really helpful. I'm quite a graphic person, I like to make my maps, things certain and in an organised manner.” Therapist, Pakistan

The importance of interdisciplinary training was raised by several participants, all from Turkey. Professionals valued the interdisciplinary approach of the web-based modules, which encouraged further exercises and case discussions within their teams. Teachers in special education appeared to have better prior understanding of interdisciplinary working.

“In other words, we always said that we should work completely interdisciplinary in the special education field, but in this training, I understood how the interdisciplinary work should be implemented in children. It raised awareness for this.” SEN Teacher, Turkey

The value of interdisciplinary training was especially acknowledged in relation to vulnerable groups such as refugee children and victims of domestic violence. This was viewed as enhancing holistic care, collaboration, and consistency across agencies. Participants wished to see this type of training extended within and across local agencies, and to involve service users.

“But it is not enough for me to understand them alone. Administrators should also be aware of this issue, so that I can help my students more. Otherwise, they may say to me ‘don't get involved in this, don’t bother’.” Teacher, Turkey

Participants from both countries, especially Pakistan, made connections between the training and the role of religion or spirituality. They related religion to identifying protective factors, formulating care plans and improving services. They hence linked counselling and religious approaches and, more broadly, psychology with Islam.

“Okay, so, adding on, the experience was remarkable! I learned a lot, and I really wish to do such more courses, because it helped me much in grooming myself as a religious teacher”. Religious scholar, Pakistan

### Theme Two: Appraisal of content, format, and delivery

Most participants were to some degree familiar with most topics addressed by module 1, although concepts such as risk/resilience were often new. They also identified aspects that extended, applied, or challenged previous knowledge. Several professionals valued the multi-dimensional approach to vulnerability, especially the socioecological framework, as they may have previously mainly focused on the child, for example, through therapy.

“One thing was very enlightening. When we think of refugee families and children, their needs, essentials, like food and shelter strike us, but nobody tends to think of their mental health needs. Everyone talks about general vulnerabilities…but with refugee children...they went through almost all of this, and thinking about them, thus highlighting this was really impressive, especially at such a platform!.” Religious scholar, Pakistan

“When I looked at the diagram here (socioecological systems) a little more carefully, I realized that there were some points I missed. I took note that I can work more complex and systematically, because sometimes we really focus on the child, and we can neglect the child’s environment.” Psychologist, Turkey

Some participants stated that they improved on practice skills such as being non-judgemental and empathic with hard-to-engage children. These views were based on theory (attachment), practical strategies, activities and videos by observing the interviewer. A ‘hands-on’ and solution-focused approach was, therefore, conducive. Practice-related questions in activities were easy to relate to and apply in their work. Participants commented on the importance of non-technical style, clarity, explanation of theories, lack of unnecessary details, stepwise learning and time-efficiency. The organisation, structure and flow of sections were important in sustaining interest. Additional proposed topics were culture and the role of media.

“And the best aspect was that, even if someone is not well-versed with the technical terms, then also s/he could understand this module very easily. No such language or difficult terminologies were used anywhere. It was comprehensive and everything was precisely explained, nor was it time-consuming.” Counsellor, Pakistan

Structuring complex concepts and information helped professionals to systematically approach their formulation of care plans. They particularly related to a holistic and hierarchical approach and felt empowered by setting specific goals. Some participants generated knowledge on trauma-informed interventions to the COVID-19 pandemic, despite the training having been designed before its outset.

“There was this goal setting explained in the course. It was very helpful to me, so I jotted it down on a notebook. It was related to planning strategy for a counselling session, especially with a vulnerable child having mental health needs, that whether it should be realistic, achievable, measurable, accountable, and short-term. It was of great advantage. I could say, it was the best part of the entire training.” Counsellor, Pakistan

Videos were positively perceived, mainly because they highlighted children’s experiences, and provided tips on interviewing skills. Flowcharts, bubbles and visual aids enhanced engagement.

“Actually, you see the point of view in every field, I think it is a very important point. Mother’s point of view, expert’s point of view, volunteer, child. I think it is very important within the scope of the multidisciplinary study programme, they impressed me very much”. NGO Worker, Turkey

There were several suggestions how digital delivery could be improved. Participants favoured more exercises, case studies, examples and creative activities. Being able to consolidate each step and revisit the modules were viewed as advantageous. Language differences could be overcome by using subtitles or dubbing.

“First of all, if I talk about the first module, the open-ended questions, there are really scary for the participant!” SEN Teacher, Turkey

Many participants favoured blended learning, through interaction with trainers and each other. This could be achieved by adding live facilitation, small group discussions, narration by trainers, interactive questions and quizzes. Having time to practice after each module could be combined with additional facilitation or face-to-face support.

“Not every employee may be interested when you give it to non-governmental organisations. He can go in and skip all the pages alone and say: ‘I looked and did’. There is no supervision situation, did he/she detect it? Did he learn? So, I think it will be much more important to be interactive. How can it be made interactive within a group using the same visuals? Maybe Zoom, etc.”. NGO Worker, Turkey

“When you’d ask my opinion about this course, I would say that this was available at per one’s comfort and ease, accessible at any moment, but I felt that, if any online facilitator would have been allocated to explain the concepts in-depth, so it would have been even better, and we’d have enjoyed a lot”. Remedial teacher, Pakistan

### Theme Three: Understanding systems

Participants shared the aspects of the training that they had already used in their practice, in the short period of a few weeks between completing the modules and attending the interview, or that they envisaged to be using in the future. Several comments related to adopting a more child-centric approach, understanding and engaging children, own cases ‘coming to life’, holistically considering their needs, and combining fun with learning to improve their wellbeing.

“It was good for me to understand which of the things I read there corresponds to the things I did in my practice, and to connect the two…I felt like it was more beneficial, because it included us and was more interactive. If it was just read, I could feel like reading a book”. Psychologist, Turkey

“After this training, I know about the risk and supporting factors, henceforth I would be working more on them; because earlier we were unconsciously not receiving these in their desired way, but this course has enlightened me on quite a few of things, which though I was aware but on a conscious level, I wasn’t working upon.” Community volunteer, Pakistan

Stepwise goal setting and action plans were commonly cited benefits. These enabled professionals to feel less overwhelmed and more in control of their care plans, by defining achievable goals and building on them over time. Several respondents viewed this as an advantage in working collaboratively with families.

“Umm...the part with step-by-step structure speaking about the action plan was really useful to me…so, if I’d design and develop my own action plan, it will help me in formulating strategies into my practice, because it would lead the plan, thereby carry the essential elements.” Counsellor, Pakistan

“I think the model will especially attract the attention of families…will be a good example in order to explain how a psychological support creates steps, and how it is more effective, because it is necessary to go to families with…concrete things.” NGO Worker, Turkey

When participants contrasted the two modules, the majority stated a preference service transformation, in that it added a new and wider perspective. They particularly related to the comprehensive scaled framework (Figure 3). Advantages included awareness of services; realising how children, families, services and society are intertwined; disciplines working together rather than in silo; agencies reflecting different aspects of a child’s life and hierarchically meeting needs. Consequently, participants made links with improvements they would like to bring in their working context. They found the mapping exercise and action plans helpful in determining local priorities. For example, they mentioned developing collaborations on the ground such as between schools and religious scholars, bringing mental health professionals into schools, and forming links with specialist services. Implications extended to NGOs developing pro-active interventions, for example, in preventing child labour. Suggestions for improvement included more hands-on activities, and a separate module on the scaled service framework.

“With this training, I saw how the mental health needs of children should be handled in the best way from the foundation to the upper stage…I do not know the basic steps and who to contact when I need help, and who I should prioritise. In the institution where I used to work, communication was established with the teachers at the school only if the parents wanted it. Other than that, there was no communication, or if there was any problem, only the family was spoken to. In this course, I have seen that not only the child, but also the family, school and all the people in the social environment when they go out should be included in the process.” SEN Teacher, Turkey

“...like, what are the strategies and what action plans should be taken, as in to set realistic goals, and execute plans which are beneficial to a child? So, I found the second module more interesting.” Educational Psychologist, Pakistan

## Discussion

In this study, we explored the perspectives of professionals from Turkey and Pakistan on which training components of a web-based foundation course could be useful for future capacity building, how these components could relate to their sociocultural contexts and working in low-resource settings and how delivery through digital platforms could augment capacity building. Participants were largely based in schools, community and religious settings. Although many participants in both countries had a first Psychology degree, they primarily worked in universal settings, especially in Pakistan. No psychiatrists were included in the sample, which possibly reflects the fragmentation between frontline and limited mental health (psychiatric) services, as reported in the interviews. This workforce and service profile broadly reflects that of MWC (Patel et al., 2018).

Overall, professionals appreciated the applicable, interdisciplinary and holistic approach of the digital training and its relevance to their practice, especially in meeting children’s complex needs. They were better acquainted with the module that related to individual casework, although its application and trauma-informed approach were new and, on some occasions, challenged previous knowledge. The latter mainly related to the wider impact of trauma and systemic changes they could initiate within their role, for which reason they found the service transformation module more innovative and empowering. Adopting a holistic approach informed by the socioecological framework in formulating assessments and care plans was perceived as particularly important in tackling multiple needs. A scaled service model was also valued, and this has been shown to lead to more efficient use of resources and joint working in MWC contexts (Sijbrandij & Acartuk, 2017). Interestingly, this model helped professionals ‘position’ child mental health along the child’s socioecology and engage families who required various material and psychosocial supports, but who would not easily seek help because of stigma.

A key finding was the appreciation of relevant theories when applied to different cultures and systems. Sometimes professionals were retrospectively made aware of the therapeutic underpinning of an intervention they had been using. Setting realistic goals is especially important in MWC where, in the face of limited specialist resources, frontline agencies can easily be overwhelmed. Indeed, participants were already using strategies learnt through the training, namely formulating holistic interventions and action plans to instigate systemic changes. For example, a recommendation in Pakistan was to link religious scholars and mental health practitioners in planning collaborative awareness initiatives. The main given reason for the appeal of this aspect was that it empowered professionals to map needs, before co-producing action plans that were achievable, short-term and measurable.

Children, young people and families can actively contribute to training and at different levels. The inclusion of videos was perceived as helpful in adopting a more child-centric approach and improving skills. Such material should be drawn from local communities. Blended learning was favoured, with digital learning, facilitation, interaction and continuity with practice. This finding is consistent with previous research in MWC (Muke et al., 2020). Exercises and case studies should be led by professionals, preferably with built-in time to practice and reflect on new knowledge and skills. Digital platforms offer opportunities to enhance the reach of training (Rahman et al., 2006); however, these should be accessible and compatible with the training format. The COVID-19 pandemic has accelerated the use of school online systems, which could be extended to community settings (Kazi et al., 2020). Working in partnership with the telecommunications industry could help in further reducing the digital and service divide.

Certain limitations of this study need to be acknowledged. The training was not intended to be comprehensive; instead, this was designed as an introduction of principles to child engagement, assessment, formulation of needs and systemic improvements; with a view to informing substantive training and capacity building. The sample was not necessarily representative of the workforce of each country, indeed of MWC globally. No child psychiatrists or health professionals participated. It would have been useful to capture the voices of children, young people and parents with lived experiences on which topics should be included and how they should be communicated during training. Professionals working in isolation, in disadvantaged communities and settings such as care homes might face additional barriers in terms of connectivity and equipment. The evaluation did not include pre- and post-training questionnaires on knowledge, skills and attitudes. Follow-up interviews would be useful in understanding whether and how lessons new knowledge was implemented and/or sustained. Crucially, future research should explore the impact of blended learning approaches.

Nevertheless, the findings of participants’ experience of completing this foundation digital training raise several implications. Linking theory with practice, providing a framework for assessment and intervention planning, adopting a holistic approach and sharing knowledge across disciplines were perceived as useful principles for future training. Crucially, a contextualised service framework to instigate systemic changes was viewed as relevant and potentially useful, although this would need implementation over a longer period. Web-based platforms can enhance access, preferably through blended learning, with ongoing facilitation and practice of new skills.

These findings can inform future capacity building, in conjunction with a range of other sources such as available evidence, policy, scoping of local needs and resources and existing training activities. Overall, child mental health capacity building in MWC should be based on a multi-level strategy. Training should be comprehensive (i.e., by including a range of problems and interventions), ongoing, supported and sustainable. It should also be tailored to different levels of needs for volunteers, universal and specialist professionals. Professional groups have their additional and unique training and development requirements. Community volunteers or paraprofessionals are a valuable resource in MWC in terms of providing awareness and engaging psychosocial programmes, hence their training, supervision and support should be designed accordingly (Van Ginneken et al., 2013). Service users and communities could contribute to preventive approaches through their unique local knowledge and relationships on the ground. They should also inform the adaptation of interventions to their sociocultural context. A parallel train-the-trainers programme would make benefits sustainable, whilst drawing on and strengthening local expertise. A cascade training model would enable local trainers to contextualise skills and competencies, whilst establishing networks and instigating systemic changes.
